# Texture-Modified Food Attitudes and Behaviours Are Culturally Patterned in Community-Dwelling Older Adults

**DOI:** 10.3390/nu18193231

**Published:** 2026-09-30

**Authors:** Verena M. H. Tan, Gary T. L. Chiah, Serene Y. L. Tan, Benjamin J. W. Chow, Meiyin Wang, Wen Wang, Jorry Dharmawan, Ives Y. Lim, Wai Yee Mah, Peng Peng Lim, Kenny B. L. Tan

**Affiliations:** 1 Health and Social Sciences Cluster, Singapore Institute of Technology, 1 Punggol Coast Road, Singapore 828608, Singapore; 2 Food, Chemical and Biotechnology Cluster, Singapore Institute of Technology, 1 Punggol Coast Road, Singapore 828608, Singaporejorry.dharmawan@singaporetech.edu.sg (J.D.); 3 Precision Health, Bioinformatics Institute, A*STAR, 30 Biopolis Street, Singapore 138671, Singapore; ives_lim@a-star.edu.sg; 4 St. Luke’s ElderCare Ltd., 461 Clementi Road, Singapore 599491, Singapore

**Keywords:** texture-modified food, food preferences, ethnicity, older adults

## Abstract

**Background/Objectives**: Texture-modified (TM) food knowledge, attitudes and behaviours (KAB) among older adults remain poorly characterized in multi-ethnic populations. The study aims: (i) to characterize food liking, consumption frequency, and TM-related KAB among community-dwelling older adults in multi-ethnic Singapore; (ii) to examine whether TM-related KAB differs by ethnicity and other demographic factors, and (iii) to evaluate associations between TM-related KAB and food liking and eating frequency. **Methods**: A cross-sectional survey was conducted from February to April 2025 among 200 older adults (≥60 years) recruited at nine active ageing centres across Singapore. Participants rated liking and consumption frequency of Chinese, Malay, Indian and Western dishes and completed KAB items related to balanced diets, nutrient importance, TM foods, and sensory attributes of soft foods. Linear mixed-effects models adjusted for age, sex, ethnicity, and body mass index were used to derive liking, frequency, and composite (liking–frequency) scores and to examine associations between KAB and food preferences. **Results**: Participants (mean age 72.7 ± 6.1 years) were 71.0% Chinese, 19.0% Malay and 10.0% Indian, with a proportion reporting dysphagia-related symptoms (5.0%) and chewing difficulties (10.0%). Compared with Chinese participants, Malay participants placed less emphasis on ease of chewing/swallowing (β = −0.860, Padj = 0.023) and soft-food aroma (β = −0.711, Padj = 0.030) but greater emphasis on maintaining a balanced diet when chewing becomes difficult (β = 0.602, Padj = 0.001). **Conclusions**: TM-related KAB varied markedly by ethnicity and cuisine but not by self-reported chewing or swallowing difficulty. These exploratory findings suggest that TM nutrition strategies tailored by ethnicity and cuisine may warrant further investigation to support healthy ageing.

## 1. Introduction

Population ageing has increased the burden of diet-related morbidity where undernutrition, sarcopenia and frailty are all common among community-dwelling older adults [1,2]. Routine nutrition screening is often recommended for early identification of malnutrition risk in older populations [3]. Systematic reviews in Asian populations showed that many older adults do not meet recommended intakes for protein, calcium, and fibre, while consuming excess saturated fat and sugars. These patterns are linked to poorer functional status and increased risk of chronic disease [1,4]. Food choice in later life is shaped by a complex interplay of physical health, oral status, sensory changes, psychological and social factors, and economic constraints, and not by nutrition guidelines alone [5]. In culturally diverse settings, such as Asia, where dietary cultures vary across countries and ethnic groups, traditional beliefs and familiarity with specific dishes further shape what older adults consider acceptable. Yet many studies still rely on Western diet indices or broad food groups that may not reflect local eating patterns [6].

Nutrition-related knowledge, attitudes, and behaviours (KAB) are one such modifiable factor. Across several cohorts of community-dwelling older adults, including in Asian settings, greater nutrition knowledge and more positive attitudes have been associated with better diet quality and healthier food choices [7,8]. However, most of this work has examined KAB as aggregate knowledge or literacy scales, linked to broad diet outcomes. Few studies have examined how specific KAB items relate to liking and intake frequency of particular dishes. In multi-ethnic Asian populations, food choice reflects a balance of health, taste, and cultural norms [5]. However, little is known about how KAB concerning balanced diets, age-related nutrition needs, or newer concepts such as texture-modified (TM) foods relates to preferences for different local cuisines [9].

Texture, particularly mouthfeel, and appearance are important determinants of appetite, intake, nutritional status, and mealtime satisfaction in older adults with and without dysphagia [10,11,12,13]. These sensory characteristics are central to the acceptance of TM foods. TM foods are increasingly recognized as an important nutritional strategy for individuals with impaired chewing or swallowing. However, most of this evidence stems from clinical and institutional settings, in individuals already diagnosed with dysphagia or already prescribed TM diets [11,14].

Dysphagia and chewing difficulties are common in older adults and are associated with malnutrition, frailty, and increased mortality [15]. Yet little is known about TM-related KAB among community-dwelling older adults, most of whom have not yet reported chewing or swallowing difficulty. This population may be well placed for proactive nutrition strategies, although whether the relevant beliefs and behaviours are already present, and how they vary, remains unexamined.

As a multi-ethnic country with substantial Chinese, Malay and Indian populations, Singapore encompasses a diverse culinary environment. Prior cohort work has identified distinct dietary patterns in older Singaporeans [1]. Existing TM research has been conducted in clinical and predominantly Western institutional settings and has not addressed this ethnic and culinary diversity. The closest related work has evaluated acceptance of a single reformulated product among older adults with pre-clinical dysphagia in a single cultural setting [16]. Three distinct gaps remain: existing KAB research has largely examined knowledge and attitudes at an aggregate level rather than in relation to specific dishes; existing TM-focused research has been conducted mainly in clinical or institutional settings rather than in community-dwelling populations; and, to our knowledge, no study has combined TM-related KAB, individual dish preferences, and multiple cuisines within a single community-dwelling population.

To address these gaps, this cross-sectional survey of community-dwelling older adults in Singapore aimed: (i) to characterize food liking, consumption frequency, and TM-related KAB among community-dwelling older adults in multi-ethnic Singapore; (ii) to examine whether TM-related KAB differs by ethnicity and other demographic factors; and (iii) to evaluate the association between TM-related KAB and food liking and eating frequency.

## 2. Materials and Methods

### 2.1. Study Design and Setting

This was a cross-sectional survey of community-dwelling older adults conducted in Singapore as part of the DIGNIFIED (Delicious, Integrative, Good Nutrition and Fulfilment in Elderly Diet) programme, a three-year multidisciplinary research programme led by the Singapore Institute of Technology and supported by the Agency for Science, Technology and Research (A*STAR). DIGNIFIED brings together food technologists and engineers, dietitians, speech therapists, sensory scientists, and chefs to develop TM foods that are nutritionally adequate, sensorially acceptable and culturally familiar to older adults in Singapore. The survey reported here was conducted to characterize the food preferences and nutrition-related KAB of older adults in the community, and to inform subsequent product development and sensory evaluation work. Data were collected from February to April 2025 using an interviewer-administered questionnaire covering sociodemographic, clinical, dietary, and knowledge, attitudes, and behaviours (KAB) measures, together with detailed ratings of liking and consumption frequency for commonly eaten local dishes. The study was approved by the Singapore Institute of Technology Institutional Review Board (SIT IRB RECAS-0497), and all participants provided written informed consent.

### 2.2. Participants and Recruitment

Eligible participants were community-dwelling adults aged 60 years and above who could provide informed consent and complete the survey in English or Mandarin. The questionnaire was administered using English and Chinese versions, both reviewed and approved by the SIT Institutional Review Board; the Chinese version was produced by translation from the approved English questionnaire. Individuals with severe cognitive impairment, including diagnosed dementia or Alzheimer’s disease, or who were otherwise unable to participate meaningfully in the interview were excluded. Recruitment was conducted in person at nine active ageing centres across Singapore, selected to span a geographic and socioeconomic range of centres. No formal stratified sampling was performed. As a broad descriptive and hypothesis-generating study, the target sample size (*N* = 200) was comparable with other community-based knowledge, attitudes and behaviours and food culture surveys conducted in this population [7,17]. The study was not formally powered for the large number of KAB-by-food-class interaction tests conducted, or for subgroup analyses, and should be regarded as exploratory.

### 2.3. Demographics and Nutritional Status

The survey consisted of two interviewer-administered instruments: the Mini Nutritional Assessment–Short Form (MNA-SF) and the survey on older adults’ food preferences. The survey collected data on age, sex, ethnicity, education, living arrangement, marital status, and self-reported chronic conditions (diabetes, heart disease, hypertension, chronic kidney disease, stroke, cancer, and high blood cholesterol), denture use, chewing difficulty and dysphagia-related symptoms. Nutritional status was assessed using MNA-SF, which classifies participants as malnourished, at risk of malnutrition, or having normal nutritional status. Participants also reported the number of main meals and between-meal snacks typically consumed per day and whether they used oral nutritional supplements. Chewing difficulty and dysphagia-related symptoms were each assessed using a single self-reported yes/no item, administered by the interviewer: “Do you have trouble chewing food?” and “Do you have any difficulty swallowing food or choking when drinking fluids?” Any affirmative response was classified as presence of the symptom; no validated dysphagia screening instrument was administered. Participants answering yes to the swallowing item were additionally asked whether they had received a formal diagnosis from a doctor or speech therapist. Participants with a prior formal diagnosis of dysphagia were not excluded from the study.

### 2.4. Food Preferences

Food preferences were assessed using a food choice inventory comprising commonly consumed Chinese, Malay, Indian and Western dishes in Singapore. Dishes were drawn mainly from the food list of the National Nutrition Survey [18]. The inventory comprised 128 dishes: 61 Chinese, 28 Malay, 29 Indian, and 10 Western. For each dish, participants rated liking on a 5-point ordinal scale as administered (1 = most favourite to 5 = do not like it at all) with an additional ‘have not eaten before’ option and reported consumption frequency on a six-category scale (never or less than once per month; 1–3 times per month; once per week; 2–4 times per week; once per day; more than one per day). Dishes were classified by cuisine, sweet versus savoury profile, and meal type (main meal versus snack). Scores were recoded for analysis so that higher values consistently indicated greater liking and more frequent consumption. Where a participant reported never having tried a given food, that item was excluded from the liking and frequency for them.

### 2.5. Knowledge, Attitudes, and Behaviours (KAB)

Nutrition-related KAB was assessed using a structured questionnaire covering general nutrition knowledge and attitudes, perceived importance of specific nutrients, confidence in healthy food choices, awareness and beliefs regarding TM foods, reasons for choosing or avoiding TM foods, and sensory priorities for soft foods. Question formats included binary yes/no items, Likert-scale ratings, ranked responses and multiple-response questions. Responses were recoded so that higher values reflected stronger endorsement, greater importance or greater exposure, as appropriate; the two willingness-to-pay items asked participants to select, from five price bands, what they would pay for a meal and for a texture-modified meal, with a higher score indicating selection of a higher price band and therefore greater willingness to pay; ‘don’t know’ and structurally skipped responses were treated as missing for the relevant analyses. Survey questions were adapted from the Food Propensity Questionnaire [19] and the National Nutrition Survey [18]. The questionnaire was delivered by trained interviewers using a standardized script. The full questionnaire is provided in Supplementary File S1.

### 2.6. Statistical Analysis

Participant characteristics were summarized as mean ± standard deviation for continuous variables and number (percentage) for categorical variables. Distributions of meal and snack frequency categories were compared using chi-square goodness-of-fit tests. Missing responses (e.g., do not know responses or structurally skipped items) were excluded from analysis.

A composite food preference score was constructed from the liking and frequency values using a rank-based weighting approach. For each outcome (liking and frequency), adjusted residuals (best linear unbiased predictions, BLUPs) were obtained adjusting for age, sex, ethnicity, BMI, and participant identifier. Adjusted values were ranked across participant-by-food observations within each outcome and transformed using a quadratic weighting function (rank^2^) to emphasize foods with consistently higher adjusted liking or consumption frequency. The rank-based weighting was used to reduce dependence on the absolute magnitude of adjusted scores while giving greater separation to foods occupying the highest ranks. The weighted ranks were then standardized to z-scores and averaged to generate the final composite food preference score. Higher weighted scores therefore reflected foods that were both more liked and more frequently consumed. As a sensitivity analysis, a simpler composite score was also calculated by averaging the standardized adjusted liking and frequency estimates without rank weighting. The two rankings were highly correlated (Pearson’s r = 0.949), with 8 of the top 10 foods shared between approaches, indicating the identification of the highest-ranked foods was broadly consistent regardless of the composite formulation.

Associations between KAB items and participant characteristics, and between KAB items and food preference measures, were examined using regression models selected according to KAB response type. Likert-scale rank, and continuous KAB outcomes were analysed using linear models or linear mixed-effects models, as appropriate. Binary and multiple-response KAB outcomes were analysed using logistic regression or logistic mixed-effects models with a binomial distribution and logit link. For mixed-effects models, participants was included as a random intercept when repeated observations were present. Binary responses coded as 1 or 2 were recoded to 0 or 1, respectively, before logistic modelling, with the lower-coded category serving as the reference category. Multiple-response items were represented as binary indicators for each selectable option. For models with food preference (adjusted liking, adjusted frequency, or composite preference score) as the dependent variable, individual KAB items were fitted as fixed effects, adjusting for age, sex, ethnicity, and BMI, with participant identifiers and food item both included as a random intercept. Models assessing effect modification by food class included interactions between KAB item and cuisine, sweet versus savoury, or meal type, with random intercepts for both participant and food item to account for repeated observations within participants and food items. Chinese cuisine, savoury foods, and main meals were used as the respective reference categories. The consistency of the composite preference score results was assessed in a sensitivity analysis using preference scores derived without demographic fixed effects. Similarly, recruitment centre was included as an additional random intercept to account for potential clustering of participants within centres as a separate additional sensitivity analysis. In both cases, no additional associations became significant, and no coefficient changed direction. The estimated coefficients were highly correlated between the two analyses (Pearson’s r = 0.99997), with a mean absolute coefficient difference of 0.00074 and a median absolute difference of approximately zero. All tests were two-sided, false discovery rate correction (Benjamini–Hochberg procedure) was applied throughout to account for multiple testing, and analyses were conducted in R (version 4.2.1).

## 3. Results

### 3.1. Participant Characteristics

A total of 200 community-dwelling older adults participated in the survey (Table 1). Participants had a mean ± SD age of 72.7 ± 6.1 years and were predominantly women (66.5%). The ethnic distribution reflected Singapore’s main ethnic groups, with 71.0% Chinese, 19.0% Malay and 10.0% Indian. Most participants had not attained tertiary education (94.5%) and lived with others (81.0%). The mean BMI was 25.2 ± 6.4 kg/m^2^. The mean MNA-SF score was 12.9 ± 1.5, indicating overall good nutritional status. Of these, 166 participants (83.0%) were classified as having normal nutritional status, 32 (16.0%) as at risk of malnutrition and 2 (1.0%) as malnourished. As MNA-SF scores were narrowly distributed near the upper end of the 0–14 scale, nutritional status was not included as a covariate in the KAB and food preference models. More than half of participants reported wearing dentures (55.5%). Self-reported dysphagia symptoms and chewing difficulty were present in 5.0% and 10.0% of participants, respectively.

### 3.2. Ethnic and Demographic Patterning of TM-Related KAB

Among the demographic factors assessed, ethnicity showed significant associations with more TM-related KAB items than any other factor (Table 2). Compared with Chinese participants, Malay participants placed lower emphasis on ease of chewing/swallowing in food choice (β = −0.860, Padj = 0.023) and on aroma as an important attribute of soft foods (β = −0.711, Padj = 0.030) but greater emphasis on the importance of a balanced diet when chewing is difficult (β = 0.602, Padj < 0.001). In a sensitivity analysis adding a random intercept for recruitment centre (nine centres, 19–27 participants each), centre accounted for 1.3% of residual variance, and the ethnicity coefficients were essentially unchanged, indicating that centre-level clustering does not account for the observed ethnic differences.

Malay participants also differed from Chinese participants on several nutrition-related items. Among the participants who ranked nutrient importance, Malay participants were more likely to rate protein as an important consideration in food choice (β = 1.097, Padj = 0.001) and less likely to rate vitamin C as important (β = −0.922, Padj = 0.030). They also reported greater willingness to pay more for food (β = 0.670, Padj = 0.035). Age was significantly associated with knowledge of TM foods: each additional year of age was associated with lower odds of reporting knowledge of TM foods (β = −0.108, Padj = 0.014).

Indian participants did not differ significantly from Chinese participants on any KAB outcome. Given the smaller Indian subgroup (*n* = 20), this should be interpreted as inconclusive rather than as evidence of no difference. No significant associations were observed between KAB outcomes and sex, BMI, self-reported chewing difficulty, dysphagia-related symptoms, or the assessed chronic health conditions in these models.

### 3.3. Eating Patterns and Nutrition Priorities

**Figure 1:**
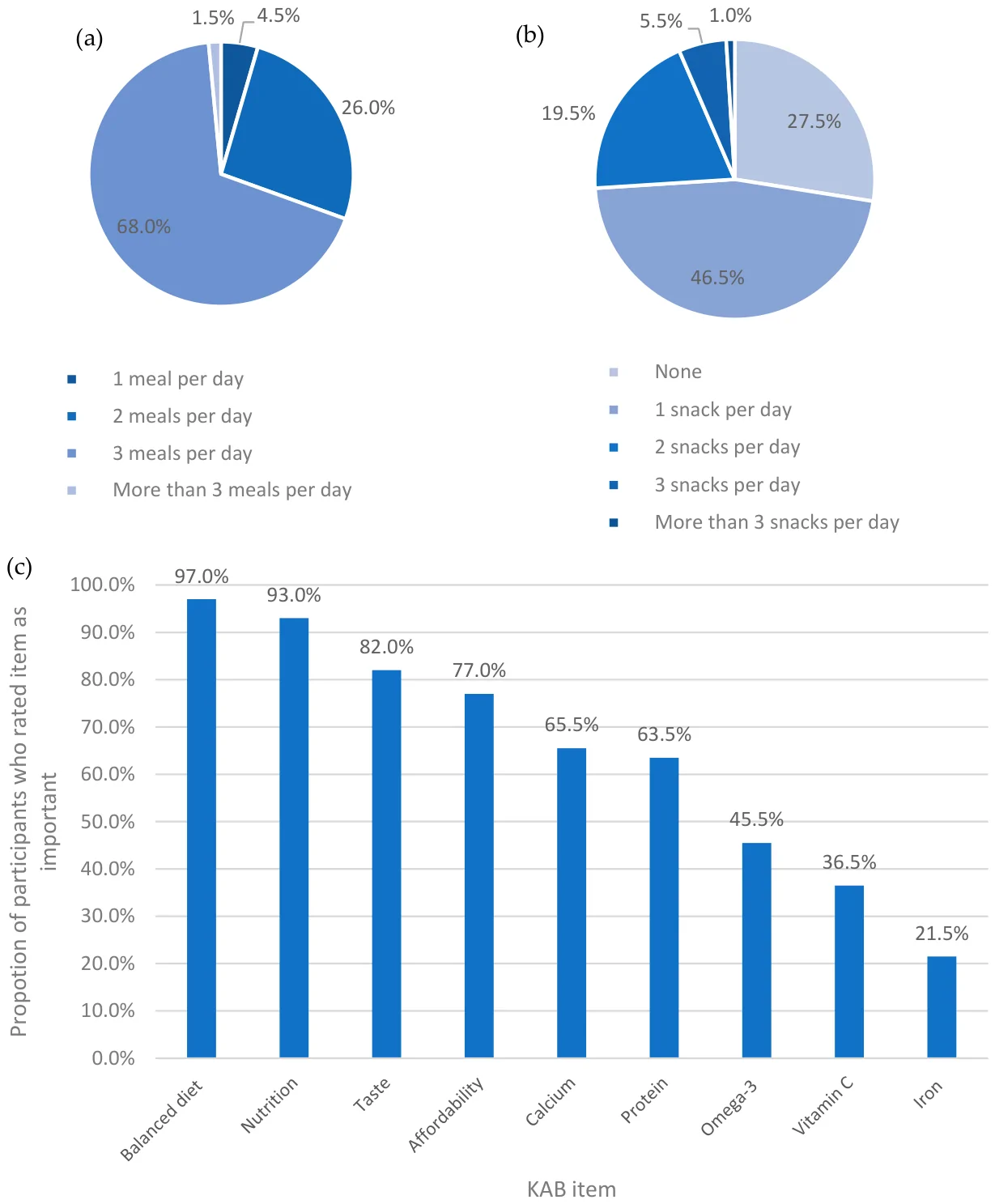
Eating patterns and importance ratings among community-dwelling older adults (*N* = 200). (**a**) Pie chart showing the proportion of main meals consumed per day. (**b**) Pie chart showing the proportion of snacks consumed per day. (**c**) Importance of each KAB item ranked in decreasing order.

Eating patterns and nutrition priorities are summarized in Figure 1a,b. Most participants reported consuming three main meals per day (68.0%), followed by two meals per day (26.0%) and one meal per day (4.5%); only 1.5% reported more than three meals per day (*p* = 0.024). Between-meal snacking was also common: 46.5% reported one snack per day, 19.5% reported two snacks per day, and 27.5% reported no daily snacks (*p* = 0.103). Oral nutritional supplement use was reported by 20.5% of participants.

Nutrition attitudes were strongly positive (Figure 1c). Overall, 97.0% rated a balanced diet as important, 93.0% rated nutritional value as important, 82.0% rated taste as important, and 77.0% rated affordability as important when choosing foods. Among 77.5% (*n* = 155) of participants who reported that nutritional needs change with age and were subsequently asked to rank nutrient importance, calcium and protein were most commonly rated as important (65.5% and 63.5%, respectively), followed by omega-3 (45.5%) and vitamin C (36.5%), whereas iron was least often prioritized (21.5%).

### 3.4. Foods with Highest Composite Preference Scores

Foods with the highest composite preference scores are presented in Table 3. Among Chinese dishes, mixed vegetable rice, fish soup, and double-boiled soups had the highest composite scores, at 1.03 ± 0.06, 0.98 ± 0.07 and 0.87 ± 0.07, respectively.

Among Malay dishes, chendol (a chilled coconut-milk dessert with pandan jelly and palm sugar) had the highest composite score (0.59 ± 0.09), followed by bo bo cha cha (a warm coconut-milk dessert with sweet potato, yam and sago; 0.26 ± 0.08) and beef rendang (beef slow-cooked in coconut milk and spices; 0.26 ±0.10).

Among Indian dishes, thosai and masala dosa (fermented rice-and-lentil crepes, plain and potato-filled, respectively) shared the highest composite score (both 0.54 ± 0.11), followed by rasam (a thin, peppery tamarind-based soup; 0.46 ± 0.16). Among Western-style items, bread ranked highly with a composite score of 0.93 ± 0.05, followed by yoghurt (0.55 ± 0.08) and biscuits (0.41 ± 0.06).

Bread and biscuits had the highest mean frequency scores among the Western-style items (2.53 and 2.61, respectively), whereas fish soup had the highest mean liking score overall (3.84). These findings indicate that a relatively small group of foods across Chinese, Malay, Indian and Western-style categories were both well liked and frequently consumed in this cohort. These composite scores reflect sensory preference and reported consumption frequency, not nutritional composition. For example, chendol, the top-ranked Malay dish, is a sugar- and coconut-milk-based dessert, and a high preference score should not be interpreted as an indicator of nutritional desirability. These foods represent the highest-ranked preferences within the predefined food list assessed in the study. For texture-modification efforts, these familiar and well-liked foods may be prioritized for texture adaptation, with further considerations and optimization on their nutritional quality.

### 3.5. KAB and Overall Food Ratings

Ninety-seven KAB associations with food ratings reached significance after false discovery rate correction. Because reporting all of these in the main text would obscure the aim of the study, Table 4 presents only those KAB items that refer directly to texture modification, chewing and swallowing, or the sensory attributes of soft foods. The remaining associations, which involve general nutrition knowledge, meal patterns and multiple-response sub-items, are reported in full in Supplementary Table S1.

Among the 16% of participants (*n* = 32) who reported having tried TM foods before, the reported frequency of TM food consumption was more strongly associated with eating frequency of Indian (β = 1.650, Padj = 0.004) and Malay dishes (β = 1.124, Padj = 0.004) than of Chinese dishes. The emphasis on choosing foods that are easier to chew or swallow showed a similar pattern, with eating frequency higher for Indian and Malay dishes than for Chinese dishes.

Two attitude items showed the opposite pattern. Participants who placed greater importance on TM food flavour familiarity ate Indian dishes less often (β = −0.584, Padj = 0.003). Those who reported greater willingness to pay for TM food consume Malay dishes less often (β = −0.579, Padj = 0.011). Two sensory items showed a positive pattern instead: greater importance placed on soft-food aroma (β = 0.527, Padj = 0.001) and on colour as a contributor to visual appeal (β = 0.456, Padj = 0.007) were each associated with higher eating frequency of Malay dishes. Mouthfeel showed a mixed pattern: independent of cuisine, greater importance placed on mouthfeel was associated with higher eating frequency across all dishes (β = 0.336, Padj = 0.033) but with lower liking specifically of Indian relative to Chinese dishes (β = −0.328, Padj = 0.014).

Among the 83 significant KAB items with food class interactions reported in Supplementary Table S1, the direction of association varied by KAB domain. Behavioural items were associated with more positive food ratings (18 out of 23 significant interactions). Attitude items were split almost evenly between positive and negative directions (21 of 40). Knowledge items showed a more heterogeneous pattern: general beliefs about balanced diets and awareness of texture-modified foods were consistently positively associated with food ratings, whereas perceived importance of specific nutrients was more often negatively associated, with calcium and iron as exceptions.

Across the 14 KAB items offering a ‘don’t know’ option, the proportion selecting it ranged from 0% to 6.5% (1.0% and 2.0% for the two general nutrition knowledge items, balanced-diet importance and healthy food choice confidence). The ‘don’t know’ response was treated as missing rather than as a substantive response category, though we acknowledge this may obscure informative variation by age or ethnicity.

### 3.6. KAB and Hedonic Food Class Preference

Three attitude items showed significant associations with hedonic food-class preference independent of cuisine (Table 4). Higher importance placed on a balanced diet was associated with greater liking of sweet relative to savoury foods (β = 0.274, Padj = 0.041) and of snacks relative to main meals (β = 0.268, Padj = 0.033). Conversely, participants who reported avoiding high-sugar foods showed greater liking for sweet relative to savoury foods (β = 0.067, Padj = 0.004; Supplementary Table S1). These interaction effects indicate that the relationship between these attitude measures and liking differed by food class; they do not by themselves establish the direction or significance of the within-class associations.

Greater importance placed on soft-food mouthfeel was associated with lower preference for sweet relative to savoury foods (β = −0.084, Padj = 0.015) and for snacks relative to main meals (β = −0.070, Padj = 0.041). In both cases, the negative interaction coefficients indicate that the estimated KAB–liking associations were lower in the named comparison class than in the corresponding reference class.

## 4. Discussion

This cross-sectional survey characterized dish-level food preferences and TM-related KAB in a multi-ethnic sample of community-dwelling older adults in Singapore, in whom self-reported dysphagia symptoms and chewing difficulty were uncommon. Among this group of older adults, TM-related KAB appeared more consistently associated with everyday food liking and eating frequency than general nutrition knowledge, and these associations differed markedly by ethnicity and cuisine. This suggests that cultural patterning of TM-related KAB is already evident among community-dwelling older adults with a low prevalence of self-reported chewing or swallowing difficulty.

Older adults in this study reported a small repertoire of dishes. This is consistent with evidence that reliance on familiar foods increases with age and remains a robust driver of food choice in later life [20,21]. Cohort data also showed that the diets of older Singaporean and Asian adults remain centred on familiar rice- and soup-based staples across sociodemographic strata [1,4,6]. The highest-scoring items shared common properties, being predominantly soft, moist, rice- or soup-based staples and coconut-milk-based sweet desserts. Because this study assessed preferences for a predefined list of dishes rather than total dietary intake, we cannot confirm what share of everyday intake these dishes represent. Nonetheless, their consistently high liking and consumption frequency in this sample can position them as culturally specific starting points for texture-modification efforts. However, it is important to note that although these dishes are candidates for texture adaptation, they are not nutritionally optimized targets for older adults. For example, chendol, the top-ranked Malay dish, is a sugar- and coconut-milk-based dessert, so its high composite score reflects familiarity and liking rather than nutritional desirability. Any translation of these dishes into menu recommendations would therefore need to consider nutritional quality separately.

Previous work has linked nutrition-related knowledge and attitudes to diet quality in older adults using composite scores and broad dietary outcomes [1,7,8]. The present findings show that specific, texture-focused KAB items are associated with liking and eating frequency at the level of individual dishes, and that these associations differ by cuisine. Emphasis on ease of chewing and swallowing, and reported TM consumption frequency among the 32 participants (16%) with prior TM experience, were both positively associated with eating frequency of Indian and Malay dishes relative to Chinese dishes.

Among sensory attributes, greater importance placed on soft-food aroma (β = 0.527) and on colour as a contributor to visual appeal (β = 0.456) was associated with higher eating frequency of Malay dishes. On the other hand, emphasis on mouthfeel was associated with higher eating frequency across all cuisines (β = 0.336). Mouthfeel is the sensory property most directly altered by texture modification [10,11,22] and this result suggests that attitudes towards the sensory qualities of soft food may be relevant to intake before chewing or swallowing difficulty develops. This is consistent with studies showing that improving sensory quality, including texture, shaping and colour, supports mealtime satisfaction and intake in older adults with dysphagia [23,24,25].

Attitudes towards TM food were not, however, consistently associated with how often TM-relevant dishes were eaten. These analyses were exploratory, given the small sample of participants reporting TM consumption frequency (*n* = 32) and should be interpreted cautiously. Eating frequency was lower among participants who placed greater importance on TM food flavour familiarity (Indian dishes, β = −0.584) and among those reporting greater willingness to pay for TM foods (Malay dishes, β = −0.579). Greater importance placed on mouthfeel was likewise associated with lower liking of Indian relative to Chinese dishes (β = −0.328). A parallel disconnect emerged between stated nutrition beliefs and hedonic preference. Participants who valued a balanced diet or reported avoiding sugar nonetheless showed stronger liking for sweet foods and snacks. This pattern is superficially consistent with the intention–behaviour gaps documented in other dietary domains, where hedonic decision factors such as taste and craving predict departures from state dietary goals [26], including among middle-aged and older adults [27], although liking rather than intake was assessed here. Both patterns are consistent with reports that older adults regard their existing dietary knowledge as sufficient and describe purchasing decisions as driven by palatability rather than health-related claims [28,29]. Older adults also hold negative views of ready-made and convenience foods even when such products would reduce preparation burden [30].

The direction of association also varied by KAB domain and by food class. Behavioural indicators were skewed positively, attitudes were evenly mixed, and nutrient-specific knowledge was more often negative, indicating that concrete, self-reported eating behaviours tracked eating frequency more closely than general knowledge or attitudes. This is consistent with evidence that knowledge and attitudes align imperfectly with reported dietary behaviour [31], and that self-reported measures in multi-ethnic Asian populations remain susceptible to social desirability bias [32]. Similar KAB profiles also translated into different preferences across sweet versus savoury items, snacks versus main meals and cuisine categories, echoing qualitative work showing that older adults reconcile health goals with enjoyment and cultural identity in ways that favour particular food classes [33].

Ethnicity showed significant associations with more TM-related KAB items than any other demographic factor assessed. Malay participants placed lower emphasis than Chinese participants on ease of chewing and swallowing and on soft-food aroma but greater emphasis on maintaining a balanced diet when chewing is difficult. Prior work in multi-ethnic Asian populations has documented comparable group-specific patterns in the perceived importance of sensory attributes and health beliefs [6,7,9], reflecting differences in cultural norms and food preparation practices.

The reasons participants gave for choosing or avoiding TM foods also varied by cuisine. Practical and hedonic reasons, including ease of consumption, personal liking and perceived nutritional value, were more strongly associated with Malay and Indian dish ratings. Sensory rejection, including disliked taste or texture and tedious preparation, was more strongly associated with Chinese dish ratings (Supplementary Table S1). This points to cuisine-specific facilitators of, and barriers to, TM adoption rather than a uniform preference for non-Chinese cuisine, and mirrors qualitative evidence that food practices among Singapore’s ethnic communities remain closely tied to identity even as cross-cultural eating is widely adopted [17]. Although Malay participants reported greater general willingness to pay for food, a willingness to pay specifically for TM food was associated with lower eating frequency of Malay dishes. Since price is commonly reported as a barrier to food choice in older adults [5], cost alone is unlikely to explain TM food uptake in this sample.

Age was inversely associated with TM food knowledge, indicating that the oldest participants were least familiar with TM concepts. As this study was cross-sectional, this association should not be interpreted as evidence of a decline in TM knowledge with age. Community-dwelling older adults have been reported to be less aware of, and less positive towards, ready-made TM foods than the speech-and-language therapists and dietitians who recommend them [34]. They are also largely unaware that nutritional requirements change with age [29,30], in keeping with the pattern here of positive general nutrition beliefs alongside weaker nutrient-specific knowledge. This gap is clinically relevant because chewing difficulty and dysphagia increase with age and are associated with malnutrition, sarcopenia, frailty and, in hospital and intermediate-care settings, higher short-term mortality [35,36]. Poorly implemented TM diets are associated with weight loss and reduced mealtime satisfaction, whereas well-presented, enriched TM foods improve intake and nutritional status [11,37], consistent with this study’s findings on soft-food aroma, mouthfeel, and visual appeal.

The barriers identified in this study appeared practical rather than informational, suggesting that skills-based support may be more useful than educational messaging—for example, demonstrating simple texture-modification techniques on dishes that older adults already consume, and providing recipes that are quick to prepare and culturally familiar. Furthermore, as engagement with TM food did not translate into greater consumption, how TM foods are presented may matter as much as how they are prepared. Hence, presenting texture modification as an adaptation of familiar dishes, rather than as a separate commercial category, may reduce the resistance older adults report towards ready-made and ‘special diet’ foods. Finally, because similar KAB profiles translate into different food-class preferences, TM strategies should consider which foods are recommended, favouring nutrient-dense main meal dishes and promoting variety across food classes and cuisines, to avoid steering older adults towards sweet, energy-dense options. Taken together, these findings indicate that TM-related nutrition strategies cannot be culturally generic, and that ethnicity and cuisine should inform how TM-related education is tailored and delivered before chewing or swallowing difficulty emerges.

The strengths of this study include the community-based sample recruited from multiple active ageing centres, the comprehensive coverage of locally relevant dishes across four cuisines, and the examination of TM-related KAB against preferences for individual dishes in a community-dwelling population with a low prevalence of self-reported chewing or swallowing difficulty. Several limitations should be acknowledged. The cross-sectional design precludes causal inference; KAB and food preferences may influence each other bidirectionally, and unmeasured factors such as income and social support may confound observed associations. All KAB measures were self-reported and may be subject to recall and social desirability bias, although the use of concrete items such as willingness to pay and specific TM beliefs may mitigate some of this. The KAB instrument has not been formally validated for this population, and the active ageing centres were not selected via formal stratified or random sampling, which may introduce selection bias not fully captured by the demographic characteristics. We mitigated this through a sensitivity analysis where centre-level clustering did not account for the observed ethnic differences. Foods that participants had never tried were excluded from the frequency and composite-score analysis, which may have affected comparisons across cuisines. The Indian subgroup was small, so null findings in this group should be regarded as inconclusive rather than as evidence of no difference. Similarly, analyses of TM consumption frequency were based on only 32 participants and should therefore be regarded as exploratory; these findings do not establish the acceptance or likely adoption of TM foods. Finally, a large number of comparisons were assessed relative to the sample size. Although false discovery rate correction was applied throughout, effect sizes should be interpreted alongside statistical significance.

## 5. Conclusions

TM-related KAB are already patterned by ethnicity and cuisine among community-dwelling older adults with a low prevalence of self-reported chewing or swallowing difficulty. These exploratory, cross-sectional findings suggest that nutrition education, menu planning, and TM product development could be tailored by ethnicity and cuisine, using these highly preferred dishes as concrete, culturally specific targets for texture-modified alternatives. Future research, including longitudinal studies, should assess whether proactive ethnicity- and cuisine-tailored TM education improves later TM acceptance compared with generic, post-diagnosis approaches, to support healthy ageing.

## Figures and Tables

**Table 1:** Sociodemographic and health characteristics of participants (*N* = 200).

Characteristic	All
Age (years), mean (SD)	72.7 (6.1)
BMI (kg/m^2^), mean (SD)	25.2 (6.4)
Ethnicity, *n* (%)	
Chinese	142 (71.0)
Malay	38 (19.0)
Indian	20 (10.0)
Education level, *n* (%)	
With tertiary education	11 (5.5)
Without tertiary education	189 (94.5)
Living conditions, *n* (%)	
Alone	38 (19.0)
With company	162 (81.0)
Marital status, *n* (%)	
Single	16 (8.0)
Married	123 (61.5)
Divorced	14 (7.0)
Widowed	47 (23.5)
MNA-SF ^1^	12.9 (1.5)
MNA-SF category, *n* (%)	
Normal	166 (83.0%)
At risk	32 (16.0%)
Malnourished	2 (1.0%)
Has dentures, *n* (%)	111 (55.5)
Dysphagia symptoms (self-reported), *n* (%)	10 (5.0)
Chewing difficulty, *n* (%)	20 (10.0)

^1^ MNA-SF: Mini Nutritional Assessment–Short Form. Dysphagia symptoms and chewing difficulty were each assessed with a single self-reported yes/no item; any affirmative response was classified as presence of the symptom. No dysphagia screen instrument was used.

**Table 2:** Demographic associations with nutrition-related knowledge, attitudes, and behaviours (KAB).

KAB Item	KAB Category	Comparison	β ^1^	SE ^2^	P_adj_
Knowledge of texture-modified foods	Knowledge	Per year of age	−0.108	0.028	0.014
Nutrient importance: protein (*n* = 155) ^3^	Knowledge	Malay vs. Chinese	1.097	0.234	0.001
Nutrient importance: vitamin C (*n* = 155) ^3^	Knowledge	Malay vs. Chinese	−0.922	0.255	0.030
Soft-food attribute: aroma	Attitude	Malay vs. Chinese	−0.711	0.199	0.030
Balanced diet importance if chewing is difficult	Attitude	Malay vs. Chinese	0.620	0.123	<0.001
Food choice: ease of chew/swallow	Behaviour	Malay vs. Chinese	−0.860	0.229	0.023
Willing to pay for food ^4^	Behaviour	Malay vs. Chinese	0.670	0.191	0.035

^1^ β: Regression coefficient; positive values indicate higher values of the KAB outcome with increasing age or relative to the specified reference category. ^2^ SE: Standard error. ^3^ Nutrient importance was assessed only among the 155 participants (77.5%) who reported that nutritional needs change with age and were subsequently asked to rank nutrient importance. ^4^ Willingness to pay was measured by asking participants to select, from five price bands, the amount they would pay for a meal; an OR above 1 indicates higher odds of selecting a higher price band. Estimates are adjusted for age, sex, ethnicity, and BMI.

**Table 3:** Top 3 foods with highest adjusted composite scores (liking and frequency combined) among older adults, by cuisine.

Cuisine	Cuisine-Specific Rank	Overall Ranking	Food	Mean Liking	SE	Mean Frequency	SE	Composite Score (Weighted z)	SE
Chinese	1	1	Mix vegetable rice ^1^	3.41	0.05	2.51	0.03	1.03	0.06
Chinese	2	2	Fish soup ^2^	3.84	0.05	2.06	0.03	0.98	0.07
Chinese	3	4	Double-boiled soups ^3^	3.66	0.05	2.12	0.03	0.87	0.07
Malay	1	5	Chendol ^4^	3.69	0.05	1.41	0.06	0.59	0.09
Malay	2	11	Bo bo cha cha ^5^	3.51	0.05	1.36	0.07	0.26	0.08
Malay	3	11	Beef rendang ^6^	3.50	0.06	1.32	0.06	0.26	0.10
Indian	1	7	Thosai ^7^	3.59	0.07	1.96	0.06	0.54	0.11
Indian	1	7	Masala dosa ^8^	3.65	0.07	1.80	0.06	0.54	0.11
Indian	3	9	Rasam ^9^	3.51	0.10	1.97	0.07	0.46	0.16
Western	1	3	Bread	3.30	0.05	2.53	0.03	0.93	0.05
Western	2	6	Yoghurt	3.35	0.05	2.43	0.04	0.55	0.08
Western	3	10	Biscuits	2.83	0.05	2.61	0.03	0.41	0.06

Liking and frequency of consumption were recoded for analysis so that higher values indicate greater liking and more frequency consumption, and means are presented on the recoded scales. Composite score (weighted z) is derived using rank-based weighting across liking and frequency, adjusted for gender, ethnicity, age, and BMI. ^1^ Mixed vegetable rice: Consists of steamed white rice topped with a choice of meat, tofu, and vegetable dishes. ^2^ Fish soup: A clear, savoury broth containing fresh fish slices, tofu, tomatoes and leafy greens and eaten with rice. ^3^ Double-boiled soups: Chinese soups that have been steamed for hours inside a large boiling vat. ^4^ Chendol: Shaved-ice dessert containing coconut milk and palm sugar. ^5^ Bo bo cha cha: A dessert containing sweet potato and yam, with chewy tapioca jelly cubes, served either warm or chilled in a sweet, pandan-infused coconut-milk broth. ^6^ Beef rendang: Slow-simmered beef in coconut milk and spice paste. ^7^ Thosai: A thin, savoury South Indian crepe made from fermented batter of rice and black grams, served hot alongside coconut chutney, tomato chutney, and lentil stew. ^8^ Masala dosa: A crispy version of thosai that is folded over, containing a stuffing of mashed potatoes spiced with mustard seeds, turmeric, onions and curry leaves. ^9^ Rasam: A South Indian soup heavily spiced with tamarind, tomatoes, black pepper, and cumin.

**Table 4:** Significant associations between knowledge, attitudes, and behaviour (KAB) items, and food ratings.

KAB Item	KAB Category	Outcome	Comparison (Reference vs. Comparison)	β	SE ^1^	P_adj_
Knowledge of TM foods	Knowledge	Frequency	Chinese vs. Malay	0.277	0.061	0.002
Knowledge of TM foods	Knowledge	Frequency	Chinese vs. Indian	0.254	0.080	0.030
TM food attribute: flavour familiarity	Attitude	Frequency	Chinese vs. Indian	−0.584	0.139	0.003
Soft-food attribute: aroma	Attitude	Frequency	Chinese vs. Malay	0.527	0.107	0.001
Visual appeal factor: colour	Attitude	Frequency	Chinese vs. Malay	0.456	0.121	0.007
Soft-food attribute: mouthfeel	Attitude	Frequency	All cuisines	0.336	0.106	0.033
Soft-food attribute: mouthfeel	Attitude	Liking	Chinese vs. Indian	−0.328	0.089	0.014
Soft-food attribute: mouthfeel ^2^	Attitude	Composite	Sweet vs. savoury	−0.084	0.024	0.015
Soft-food attribute: mouthfeel ^2^	Attitude	Composite	Snack vs. main meal	−0.070	0.022	0.041
Willingness to pay: TM food ^3^	Attitude	Frequency	Chinese vs. Malay	−0.579	0.162	0.011
TM consumption frequency (*n* = 32) ^4^	Behaviour	Frequency	Chinese vs. Indian	1.650	0.415	0.004
TM consumption frequency (*n* = 32) ^4^	Behaviour	Frequency	Chinese vs. Malay	1.124	0.283	0.004
Food choice: ease of chewing/swallowing	Behaviour	Frequency	Chinese vs. Indian	0.653	0.156	0.003
Food choice: ease of chewing/swallowing	Behaviour	Frequency	Chinese vs. Malay	0.503	0.120	0.003

^1^ SE: Standard error. ^2^ Association with the adjusted composite score. ^3^ Willingness to pay was measured by asking participants to select, from five price bands, the amount they would pay for a texture-modified meal; a higher score indicates a higher price band. ^4^ TM consumption frequency was assessed only among 32 participants (16%) who reported having previously tried texture-modified foods. Linear mixed-effects models with random intercepts for participant and food item, adjusted for age, sex, ethnicity, and BMI, were employed. β coefficients for food-class interactions represent the difference in the association between the KAB item and the specified food-rating outcome between the named comparison class and the reference class. They do not represent within-class associations. Positive β values indicate a stronger association in the comparison class than in the reference class, whereas negative β values indicate a weaker association. *p* values were adjusted using the Benjamini–Hochberg procedure within the corresponding food-rating outcome. Table 4 presents the significant findings highlighted in the main text; additional significant findings are reported in Supplementary Table S1.

## Data Availability

The original contributions presented in this study are included in the article/Supplementary Material. Further inquiries can be directed to the corresponding author.

## Supplementary Materials

The following supporting information can be downloaded at https://www.mdpi.com/article/10.3390/nu18193231/s1. Table S1: Significant associations between nutrition-related knowledge, attitudes and behaviours (KAB) items and food-class preference; File S1: Questionnaire on Food Preferences. References [19,38,39,40,41,42] are cited in the Supplementary Materials.
