# Supplementary material for: Texture-Modified Food Attitudes and Behaviours Are Culturally Patterned in Community-Dwelling Older Adults
Source: Nutrients. 2026 Sep 30;18(19):3231. doi: 10.3390/nu18193231 (PMC13666978; doi:10.3390/nu18193231)
Supplement: Supplementary file 1 [file nutrients-18-03231-s001.zip › File S1. Questionnaire on Food Preferences.pdf]

Name of interviewer: _________________________ Date of interview: ____________________

Questionnaire on Food Preferences

Section A: Demographics

Gender: Female/ Male (please circle) **Ethnicity:** Chinese/ Malay/ Indian (please circle)

Date of Birth:

_____(day) /___(month)/_____(year)

**Height:** _________ (cm)<br><br>**Weight:** _________ (Kg)	**1) What is your highest educational attainment?** (Please choose one option)<br><br>☐ Primary School<br>☐ Secondary School<br>☐ Diploma<br>☐ Bachelor's degree<br>☐ Postgraduate<br>Others: ______________
**2) Who are you currently living with?**<br><br>☐ Alone<br>☐ With spouse<br>☐ With children<br>☐ With children and spouse<br>☐ With live-in helper<br>Others: ______________	**3) Marital Status:**<br><br>☐ Single<br>☐ Married<br>☐ Divorced<br>☐ Widowed
**4) Do you have any health issues**: (you may choose more than one)<br><br>☐ Diabetes<br>☐ Heart disease<br>☐ Hypertension<br>☐ Chronic kidney disease<br>☐ Stroke<br>☐ Cancer<br>☐ High Blood Cholesterol<br>Others: ___________________	**5) Are you currently wearing dentures**<br><br>Yes No<br><br>If Yes, describe your current use of dentures: (Please choose one option)<br><br>☐ Full Upper Dentures<br>☐ Partial Upper Dentures<br>☐ Full Lower Dentures<br>☐ Partial Lower Dentures<br>☐ Full Upper and Lower Dentures<br>Others: ____________________

6) **What best described your fitting of Denture?** (Please choose one option only)

☐ Securely fit ☐ Slightly loose ☐Loose fit ☐ Very loose fit

7) **Do you have any difficulty swallowing food or choking when drinking fluids?** Yes No

If Yes, have you been formally diagnosed by a doctor or a speech therapist?

Yes ______________________(what was the diagnosis?) No

8) Do you have trouble chewing food? Yes No

If Yes, how do you make chewing food easier? Please tick your options (you may choose more than one)

- ☐ Cut food into smaller pieces
- ☐ Mash or puree it
- ☐ Use gravy, sauces or broth to add moisture to food
- ☐ Use moist-heat cooking methods e.g. steamed/ boiled
- ☐ Avoid hard chewy food

9) Have you ever received advice from a healthcare professional about including textured modified foods into your diet?

Yes No

==

Name of interviewer: ________________________ Date of interview: ___________________

KNOWLEDGE, ATTITUDES, BEHAVIOURS AND FOOD PREFERENCES

Sections B: Knowledge (please select one option only)

1) Is it important to maintain a balanced diet as you age?

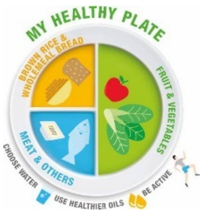

☐ Very Important
☐ Important
☐ Somewhat Important
☐ Not Important
☐ Don't know

2) Do nutritional needs change as we age?

☐ Yes (go to Q3) ☐ No (skip to Q4)

3) Which of the following nutrients do you think are important to age well? Rank them in order of importance (1 being the most important and 5 being the least important)

______ Calcium
______ Protein
______ Vitamin C
______ Omega 3- fatty acids
______ Iron

4) Are you confident to choose healthy whole foods (e.g. wholegrains, fruits, vegetables, lean meat/ poultry/fish, dairy products) as you age?

☐ Very Confident
☐ Confident
☐ Somewhat Confident
☐ Not Confident
☐ Don't know

5) Do you know what are texture modified foods?

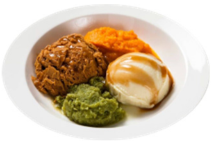

IDDSI Level 4 (Pureed)

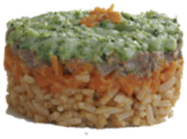

IDDSI Level 5 (Minced & Moist)

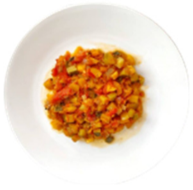

IDDSI Level 6 (Soft & Bite Sized)

☐ Yes (go to Q6) ☐ No

6) What do you think are the benefits of consuming textured modified foods (minced and moist)? (Select all that apply)

☐ Easier to chew and swallow
☐ Better nutrients absorption
☐ Improved digestion
☐ No benefits
☐ Don't know

<u>Section C: Attitudes</u> (please select one option only)

1) If you have chewing/ swallowing difficulties, how important is it to maintain a balanced diet as you age?

- ☐ Very Important
- ☐ Important
- ☐ Somewhat Important
- ☐ Not Important
- ☐ Don’t know

2) If you have chewing/ swallowing difficulties, how confident are you to choose nutritious foods (wholegrains, fruits, vegetables, lean meat/ poultry/ fish, dairy products) that meet your dietary needs as you age?

- ☐ Very Confident
- ☐ Confident
- ☐ Somewhat Confident
- ☐ Not Confident
- ☐ Don’t know

3) If you have chewing or swallowing difficulties, would you choose to consume texture-modified foods? (Regardless of whether you have tried them before.)

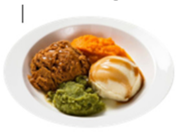

IDDSI Level 4 (Pureed)

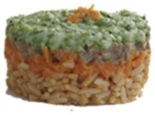

IDDSI Level 5 (Minced & Moist)

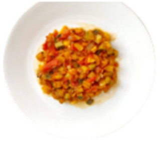

IDDSI Level 6 (Soft & Bite Sized)

☐ Yes (go to Q4) ☐ No (go to Q5)

4) What are the reasons you would choose to eat textured modified foods? (Select all that apply)

- ☐ No choice, recommendations from healthcare professional due to medical condition
- ☐ Easy to consume
- ☐ Easy to prepare
- ☐ Not confident in eating regular textured food
- ☐ Personal liking
- ☐ Nutritious
- ☐ Tasty

5) What are the reasons you would choose NOT to eat textured modified foods? (Select all that apply)

- ☐ Dislike the texture
- ☐ Dislike the taste
- ☐ Does not look appetizing
- ☐ Tedious to prepare
- ☐ Does not know how to prepare

6) If textured modified Asian food products (minced and moist) were available in the supermarket, would you consider buying them for your meals?

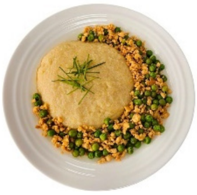

Mui Fan

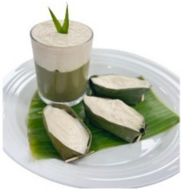

Kueh Tepung Pelita

☐ Yes ☐ No (go to Q7)

7) What are some reasons you will NOT buy textured modified Asian food products (minced and moist) for your meals? (Select all that apply)

☐ Not sure how they will taste
☐ Might be expensive
☐ Difficult to prepare (e.g. no microwave)
☐ May not be nutritious
Others: _________________________________

8) Do you believe that consuming textured modified foods can improve your ability to eat better?

☐ Strongly Agree
☐ Agree
☐ Disagree
☐ Strongly Disagree
☐ Don't know

Section D: Behaviours (please select one option only)

1) I prefer to eat home-cooked meals rather than eating out or ready-made meals

☐ Strongly Agree
☐ Agree
☐ Disagree
☐ Strongly Disagree
☐ Don't know

2) Are there any foods you avoid/limit due to health reasons? (Select all that apply)

☐ High in salt, e.g. ham/sausage/canned food/soy sauce
☐ High in sugar, e.g. candies/ desserts/ fruit juices/sugary drinks
☐ High in fats, e.g. oil/ butter/ ghee/fried foods/ pastries
☐ Dairy products e.g. milk/ cheese/ yogurt/ cream
☐ I do not avoid any foods
☐ Others: __________________

3) I choose foods that are easy to chew and swallow for my daily meals

☐ Always
☐ Often
☐ Sometimes
☐ Rarely
☐ Never

4) Have you tried textured modified foods before?

Yes _____________________(indicate IDDSI level) No (skip to Q8)

5) Did you like the textured modified foods you tried?

☐ Yes
☐ No

If yes/ no, Why? ____

6) How often do you consume textured modified foods as part of your regular diet?

☐ Daily
☐ Weekly
☐ Monthly
☐ Rarely
☐ Never

7) What challenges do you face if you need to prepare textured modified foods? (Select all that apply)

☐ Unsure about how to prepare the correct texture
☐ Lack of cooking equipment at home
☐ Does not know how to cook
☐ Takes too much time to prepare
☐ No challenges

8) How many main meals (e.g. breakfast, lunch, dinner) do you typically eat per day? For interviewer: at least 2 CHO exchange= main meals

☐ 1 meal per day
☐ 2 meals per day
☐ 3 meals per day
☐ More than 3 meals per day

9) How many mid-meal snacks (a small portion of food that is smaller than a main meal) do you eat per day? For interviewer= < 2 CHO exchange = snacks

☐ 1 snack per day
☐ 2 snacks per day
☐ 3 snacks per day
☐ More than 3 snacks per day
☐ None

10) **Are you currently taking any oral nutritional supplements? Some examples include:**

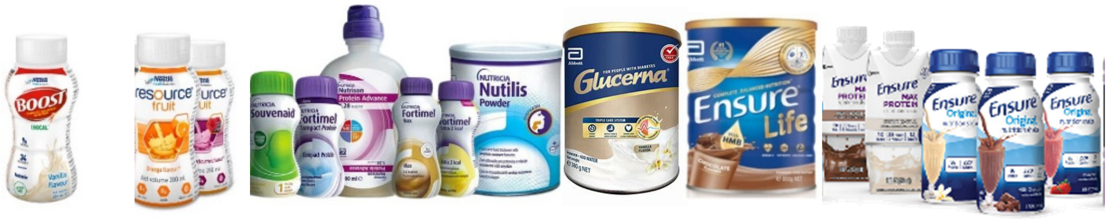

☐ Yes ☐ No

If Yes, please provide the name and quantity:____

11) **Would you be interested to attend a workshop for ageing well?**

☐ Yes ☐ No

<u>Section E: Food Preferences</u> (please select one option only)

1) **How important is the taste of the food you eat on a typical day?**

- ☐ Very Important
- ☐ Important
- ☐ Somewhat Important
- ☐ Not Important
- ☐ Don't know

2) **How important is the nutritional value of the food you eat on a typical day?**

- ☐ Very Important
- ☐ Important
- ☐ Somewhat Important
- ☐ Not Important
- ☐ Don't know

3) **How important is it to you that the food you eat on a typical day is affordable?**

- ☐ Very Important
- ☐ Important
- ☐ Somewhat Important
- ☐ Not Important
- ☐ Don't know

4) **How much are you willing to pay for a meal?**

- ☐ < $5
- ☐ $5-6
- ☐ $7-8
- ☐ $9-10
- ☐ >$10

5) **How much are you willing to pay for a textured modified meal?**

☐ < $5
☐ $5-6
☐ $7-8
☐ $9-10
☐ >$10

6) **How important is it to you that the textured modified foods (minced and moist) you eat have flavours that you are familiar to? (e.g. nasi lemak, laksa, chicken rice)**

☐ Very Important
☐ Important
☐ Somewhat Important
☐ Not Important
☐ Don't know

7) **How important is it to you that the textured modified foods (minced and moist) you eat look like the traditional dishes you are familiar with? (e.g. nasi lemak, laksa, chicken rice)**

☐ Very Important
☐ Important
☐ Somewhat Important
☐ Not Important
☐ Don't know

Name of interviewer: ______________________ Date of interview: ________________

Section F

Attitudes on Textured Modified Foods (Minced & Moist, Pureed)

The pictures below are some examples of minced and moist/ pureed food

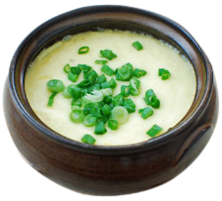<br>**Steamed Egg**<br>(Minced and moist/ pureed)	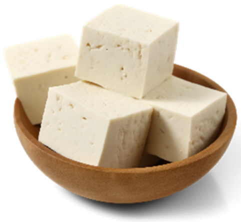<br>**Steamed Tofu**<br>(Minced and moist)	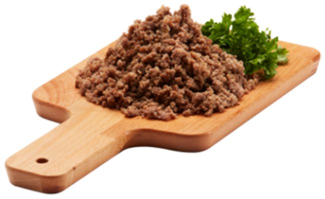<br>**Minced Meat**<br>(Minced and moist)
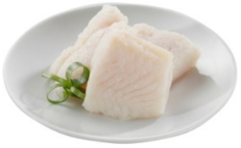<br>**Steamed Fish**<br>(Minced and moist)	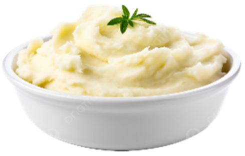<br>**Mashed Potatoes**<br>(Minced and moist/ pureed)	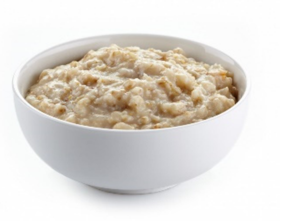<br>**Oats**<br>(Minced and moist)
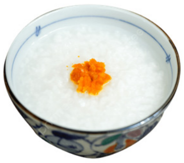<br>**Porridge**<br>(Minced and moist/ pureed)	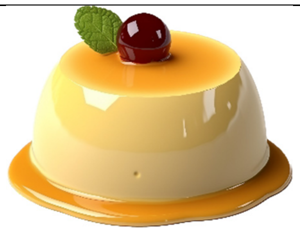<br>**Pudding**<br>(Minced and moist/ pureed)	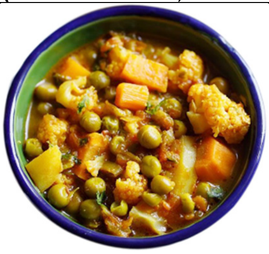<br>**Soft Cut Vegetables**<br>(Minced and moist)

1) Which of the following would make soft food more palatable to you? Please rank them in order of importance, with 1 being the most important and 4 being the least important.

**Aroma**<br>The smell of the food.	**Mouthfeel**<br>The sensation of the food in your mouth while eating, including its texture, such as smooth, creamy, crunchy, or chewy	**Appearance**<br>The attractiveness of the food's appearance, including its colour, shape, and presentation.	**Taste**<br>The taste of the food, encompassing basic flavours such as sweet, salty, sour, bitter, and umami (Savory).

2) Regarding appearance, which of the following factors would enhance the visual appeal of the food for you? Please rank them in order of importance, with 1 being the most important and 4 being the least important.

**Colour**<br>The colours and tones of the food.	**Shape**<br>The form or cut of the food.	**Garnishes/ Toppings**<br>Additional decorative elements added to food, such as herbs, spices, or condiments.	**Plating**<br>The arrangement of food on the plate

Subject ID: ___________________ DIGNIFIED PROGRAMME

Name of interviewer: _________________________ Date of interview: ____________________

FOOD PREFERENCES QUESTIONNAIRE

The following food frequency table is designed to estimate your **usual eating pattern**.

The boxes include monthly, weekly and daily categories:
Never or (about) less than once a month (<1 per /month)
1-3 (times) per month
1 per week
2-4 (times) per week
1 time per day
>1 per day

For example, foods you never or rarely eat would be checked “never”. A food eaten only a few times during a particular season would also be checked “never”. Foods eaten only a few times during the week or eaten a few times on the weekend would be checked “2-4 times a week”. A food eaten more than once a day would checked as “2-3 times a day”.

For food liking, the ranking is as follows: 1 represents "most favourite," 5 means "do not like it at all.

	Food Frequency							Food Liking					
	**Food Products**	**Never or <1 per month**	**1-3 times per month**	**1 time per week**	**2-4 times per week**	**1 time per day**	**>1 per day**	**1**	**2**	**3**	**4**	**5**	**Have not eaten before**
1	Biscuits 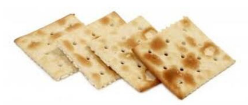												
2	Bread 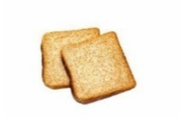												
3	Cakes 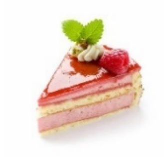												
4	Chicken sandwich 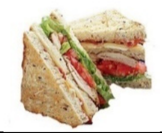												
5	Chocolate 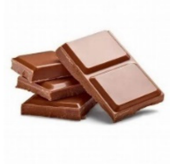												
6	Egg sandwich 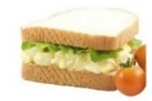												

Subject ID: ___________________ DIGNIFIED PROGRAMME

Name of interviewer: _________________________ Date of interview: ____________________

	Food Frequency							Food Liking					
	**Food Products**	**Never or <1 per month**	**1-3 times per month**	**1 time per week**	**2-4 times per week**	**1 time per day**	**>1 per day**	**1**	**2**	**3**	**4**	**5**	**Have not eaten before**
7	Hot dog bun 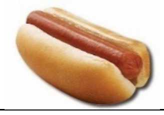												
8	Kaya Toast 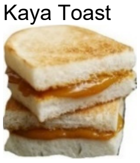												
9	Pau with meat fillings 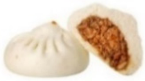												
10	Rojak 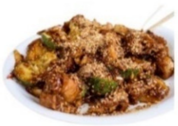												
11	Satay 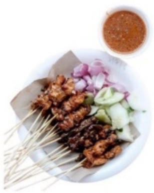												
12	Traditional ice cream (bread/ biscuits) 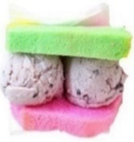												
13	Tuna sandwich 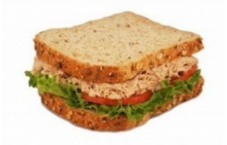												
14	Yogurt 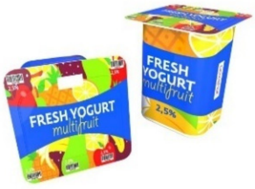												

Name of interviewer: ______________________________________ Date of interview: ____________________

Chinese Food													
	Food Frequency							Food Liking					
	**Food Products**	**Never or <1 per month**	**1-3 times per month**	**1 time per week**	**2-4 times per week**	**1 time per day**	**>1 times per day**	**1**	**2**	**3**	**4**	**5**	**Have not eaten before**
1	ABC soup/ lotus root (double boiled soups) 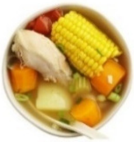												
2	Bak chor mee 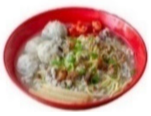												
3	Bak ku teh 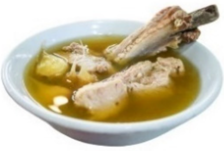												
4	Butterfly fritters/ donuts 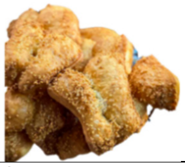												
5	Carrot cake 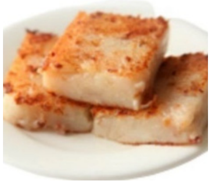												
6	Char kway teow 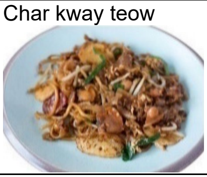												
7	Chee Cheong Fun 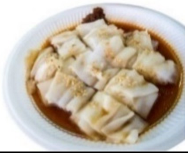												
8	Cheng teng 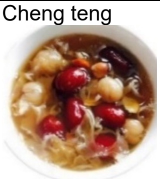												
9	Chicken rice 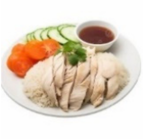												

Subject ID: ___________________ DIGNIFIED PROGRAMME

Name of interviewer: _________________________ Date of interview: ____________________

Chinese Food													
	**Food Frequency**							**Food Liking**					
	**Food Products**	**Never or <1 per month**	**1-3 times per month**	**1 time per week**	**2-4 times per week**	**1 time per day**	**>1 times per day**	**1**	**2**	**3**	**4**	**5**	**Have not eaten before**
10	Chinese Vegetable curry 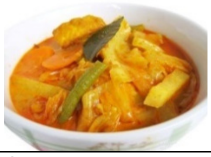												
11	Chwee Kueh 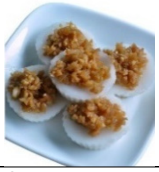												
12	Claypot rice 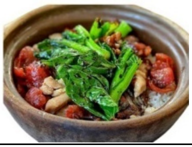												
13	Curry puff 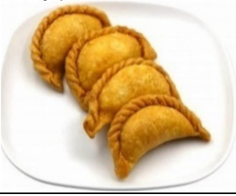												
14	Da mai dessert 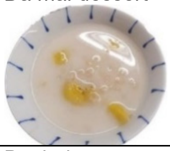												
15	Duck rice 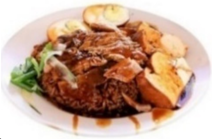												
16	Dumpling (Zhong zi) 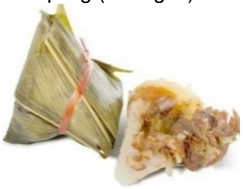												
17	Dumpling noodles 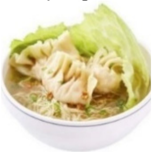												
18	Egg tarts 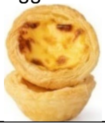												

Chinese Food													
	Food Frequency							Food Liking					
	**Food Products**	**Never or <1 per month**	**1-3 times per month**	**1 time per week**	**2-4 times per week**	**1 time per day**	**>1 times per day**	**1**	**2**	**3**	**4**	**5**	**Have not eaten before**
19	Fish curry 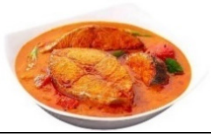												
20	Fish soup 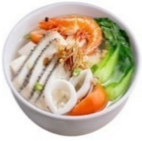												
21	Fishball noodles 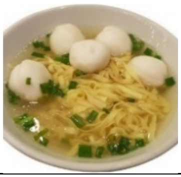												
22	Fried carrot cake 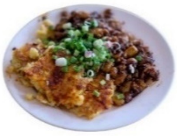												
23	Fried fishball 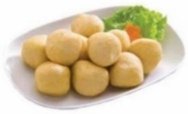												
24	Economic beehoon 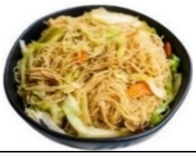												
25	Economic noodles 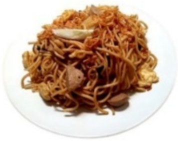												
26	Fried rice 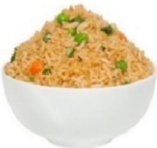												
27	Green bean soup 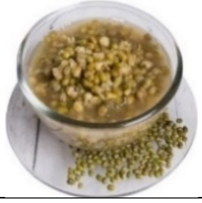												

Name of interviewer: _________________________ Date of interview: ____________________

Chinese Food													
	Food Frequency							Food Liking					
	**Food Products**	**Never or <1 per month**	**1-3 times per month**	**1 time per week**	**2-4 times per week**	**1 time per day**	**>1 times per day**	**1**	**2**	**3**	**4**	**5**	**Have not eaten before**
28	Hokkien Mee 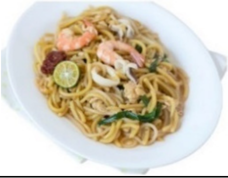												
29	Hor fan 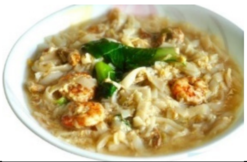												
30	Kway chap 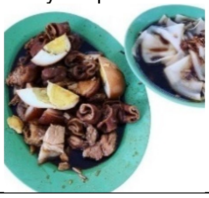												
31	Laksa 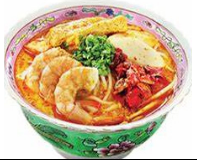												
32	Lei cha 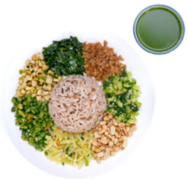												
33	Lor mai kai 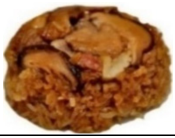												
34	Lor mee 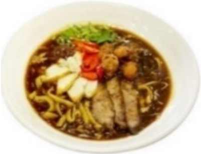												
35	Mee jiang kueh 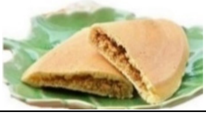												
36	Mix veg rice 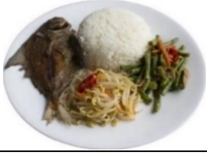												

Subject ID: ___________________ DIGNIFIED PROGRAMME

Name of interviewer: _________________________ Date of interview: ____________________

Chinese Food													
	Food Frequency							Food Liking					
	**Food Products**	**Never or <1 per month**	**1-3 times per month**	**1 time per week**	**2-4 times per week**	**1 time per day**	**>1 times per day**	**1**	**2**	**3**	**4**	**5**	**Have not eaten before**
37	Mooncake 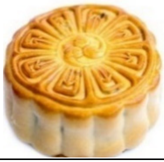												
38	Muah chee 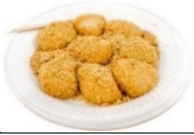												
39	Mui fan 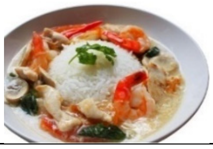												
40	Pineapple tarts 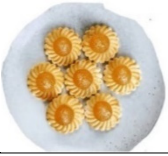												
41	Popiah 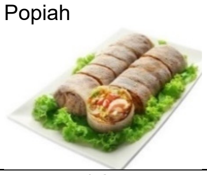												
42	Porridge/ Congee 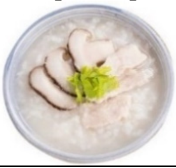												
43	Prawn noodles 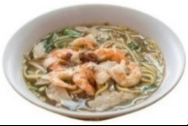												
44	Red bean pau 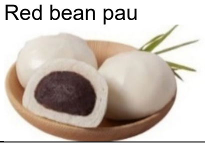												
45	Red bean soup 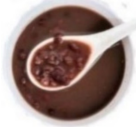												
46	Roasted meat rice 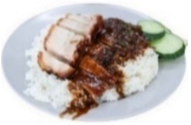												

Name of interviewer: _________________________ Date of interview: ____________________

Chinese Food													
	Food Frequency							Food Liking					
	**Food Products**	**Never or <1 per month**	**1-3 times per month**	**1 time per week**	**2-4 times per week**	**1 time per day**	**>1 times per day**	**1**	**2**	**3**	**4**	**5**	**Have not eaten before**
47	Siew mai 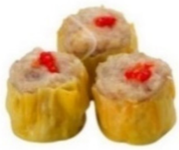												
48	Soft- boiled eggs 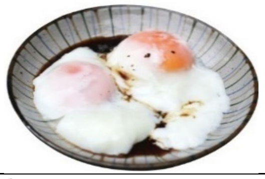												
49	Soon Kueh 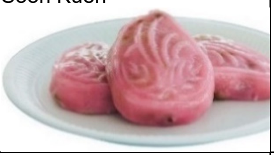												
50	Soy beancurd 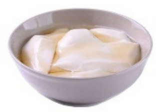												
51	Steamed bun 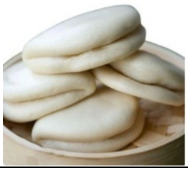												
52	Tang Yuan 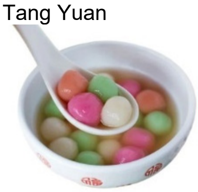												
53	Tau suan 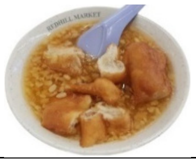												
54	Wonton noodles 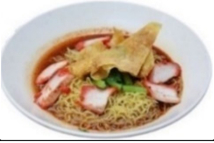												
55	Yam cake 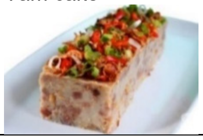												

Chinese Food													
	Food Frequency							Food Liking					
	**Food Products**	**Never or <1 per month**	**1-3 times per month**	**1 time per week**	**2-4 times per week**	**1 time per day**	**>1 times per day**	**1**	**2**	**3**	**4**	**5**	**Have not eaten before**
56	Yong Tau Fu 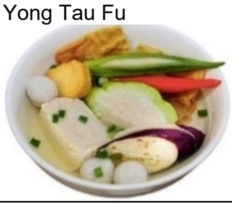												
57	You tiao 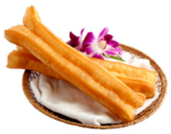												

Subject ID: ___________________ DIGNIFIED PROGRAMME

Name of interviewer: _________________________ Date of interview: ____________________

Malay Food													
	Food Frequency							Food Liking					
	**Food Products**	**Never or <1 per month**	**1-3 times per month**	**1 time per week**	**2-4 times per week**	**1 time per day**	**>1 time per day**	**1**	**2**	**3**	**4**	**5**	**Have not eaten before**
1	Ayam Goreng 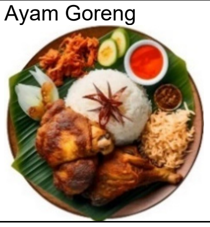												
2	Ayam masak merah 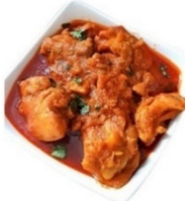												
3	Beef rendang 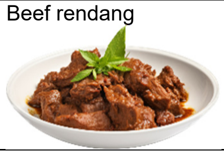												
4	Bo bo cha cha 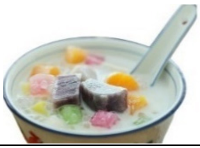												
5	Chendol 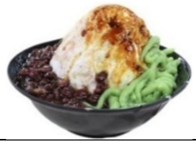												
6	Epok epok 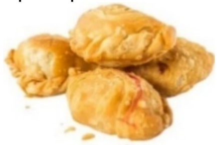												
7	Gado gado 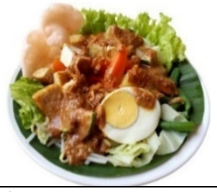												
8	Goreng pisang 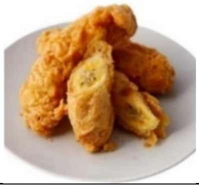												
9	Ice Kachang 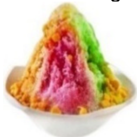												

Subject ID: ___________________ DIGNIFIED PROGRAMME

Name of interviewer: _________________________ Date of interview: ____________________

Malay Food													
	**Food Frequency**							**Food Liking**					
	**Food Products**	**Never or <1 per month**	**1-3 times per month**	**1 time per week**	**2-4 times per week**	**1 time per day**	**>1 time per day**	**1**	**2**	**3**	**4**	**5**	**Have not eaten before**
10	Ikan Bakar 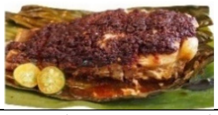												
11	Kueh (e.g kueh salat/ kueh lapis/ kueh dadar etc) 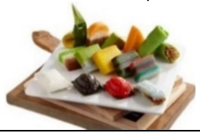												
12	Lontong 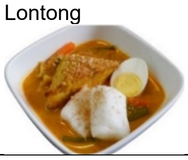												
13	Mee goreng 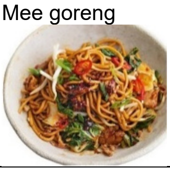												
14	Mee rebus 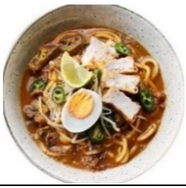												
15	Mee Siam 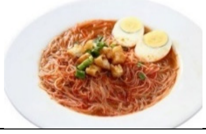												
16	Mee soto 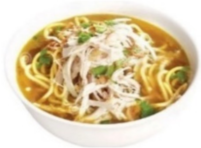												
17	Nasi Goreng 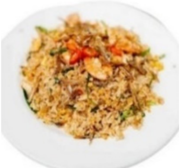												
18	Nasi Lemak 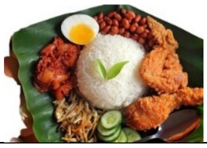												
19	Nasi padang 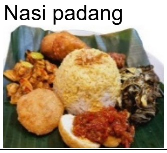												

Subject ID: ___________________ DIGNIFIED PROGRAMME

Name of interviewer: _________________________ Date of interview: ____________________

Malay Food														
	**Food Frequency**							**Food Liking**					
	**Food Products**	**Never or <1 per month**	**1-3 times per month**	**1 time per week**	**2-4 times per week**	**1 time per day**	**>1 time per day**	**1**	**2**	**3**	**4**	**5**	**Have not eaten before**
20	Ondeh- Ondeh 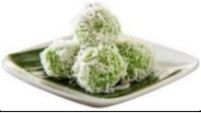												
21	Pulut Hitam 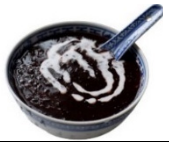												
22	Putu piring 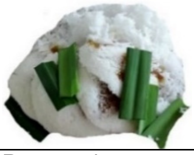												
23	Rempeyek 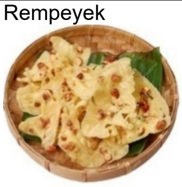												
24	Roti John 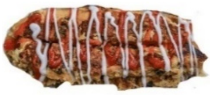												
25	Sambal goreng 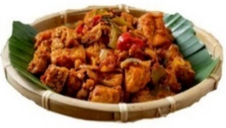												
26	Sambal Udang 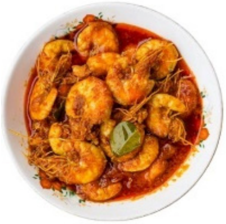												
27	Sayur lodeh 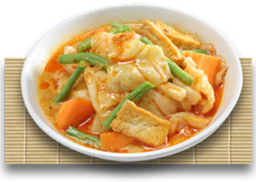												
28	Serunding 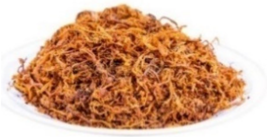												

Subject ID: ___________________ DIGNIFIED PROGRAMME

Name of interviewer: _________________________ Date of interview: ____________________

Indian Food													
	**Food Frequency**							**Food Liking**					
	**Food Products**	**Never or <1 per month**	**1-3 times per month**	**1 time per week**	**2-4 times per week**	**1 time per day**	**>1 time per day**	**1**	**2**	**3**	**4**	**5**	**Have not eaten before**
1	Aloo gabi 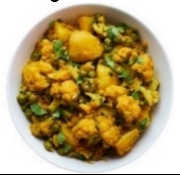												
2	Appam 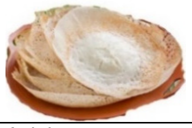												
3	Avial 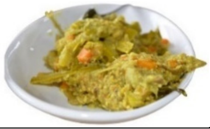												
4	Butter chicken 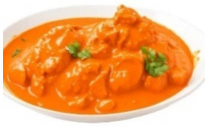												
5	Chapatti 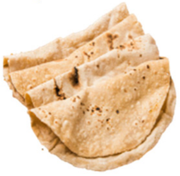												
6	Chettinad chicken 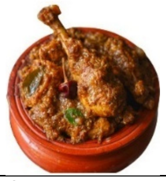												
7	Chutney (tomatoes/ coconut) 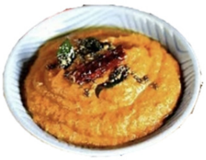												
8	Fishhead curry 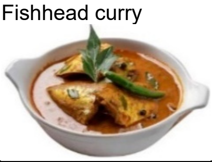												
9	Idli 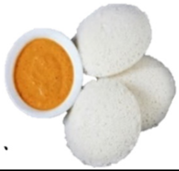												

Indian Food													
	Food Frequency							Food Liking					
	**Food Products**	**Never or <1 per month**	**1-3 times per month**	**1 time per week**	**2-4 times per week**	**1 time per day**	**>1 time per day**	**1**	**2**	**3**	**4**	**5**	**Have not eaten before**
10	Indian vegetable curry 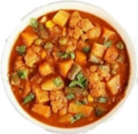												
11	Kara Kuzhambu 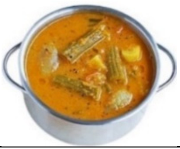												
12	Kootu 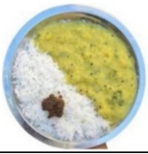												
13	Kuzhi Paniyaram 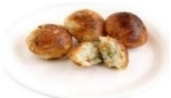												
14	Ladoo 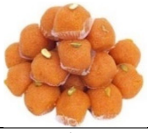												
15	Lentils/ Dhal 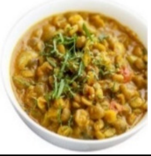												
16	Masala dosa 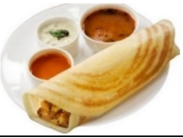												
17	Murtabak 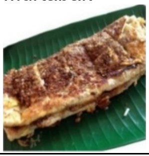												
18	Naan 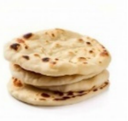												
19	Nasi Biryani 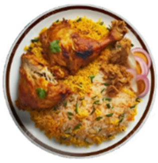												

Name of interviewer: _________________________ Date of interview: ____________________

Indian Food													
	**Food Frequency**							**Food Liking**					
	**Food Products**	**Never or <1 per month**	**1-3 times per month**	**1 time per week**	**2-4 times per week**	**1 time per day**	**>1 time per day**	**1**	**2**	**3**	**4**	**5**	**Have not eaten before**
20	Pongal 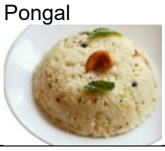												
21	Puttu Mayam 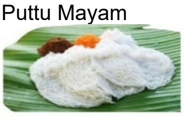												
22	Rasam 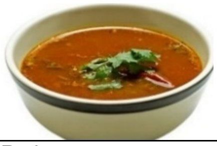												
23	Roti prata 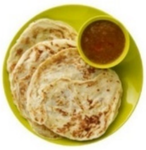												
24	Samosa 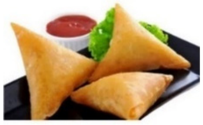												
25	Tandoori chicken 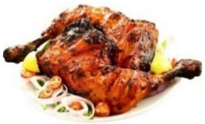												
26	Thosai 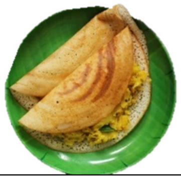												
27	Upma 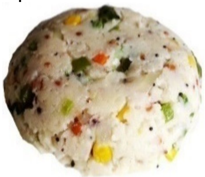												
28	Uttapam 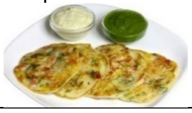												
29	Vadai 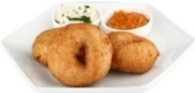												

If you selected mixed vegetable rice/ Nasi Padang, please respond to the following questions:

Most common food options:	Carbohydrates (e.g. rice/ porridge)	Protein (e.g. steamed fish/sweet and sour chicken)	Fibre (e.g. egg plant/ ladies' fingers)	Gravy (e.g. curry/ dark sauce/ asam pedas)
Variation 1:				
Variation 2:				

Questions were adapted from the Food Propensity Questionnaire, National Nutrition Survey [6] [7]

Thank you for your participation!

References

[1] Kim, C.-O. (2016). Food choice patterns among frail older adults: The associations between social network, food choice values, and diet quality. Appetite, 96, 116–121. https://doi.org/10.1016/j.appet.2015.09.015

[2] Kamphuis, C. B., de Bekker-Grob, E. W., & van Lenthe, F. J. (2015). Factors affecting food choices of older adults from high and low socioeconomic groups: a discrete choice experiment. The American Journal of Clinical Nutrition, 101(4), 768–774. https://doi.org/10.3945/ajcn.114.096776

[3] Roininen, K., Tuorila, H., Zandstra, E. H., de Graaf, C., Vehkalahti, K., Stubenitsky, K., & Meland, D. J. (2001). Differences in health and taste attitudes and reported behaviour among Finnish, Dutch and British consumers: A cross-national validation of the Health and Taste Attitude Scales (HTAS). Appetite, 37(1), 27-38. https://doi.org/10.1006/appe.2001.0422

[4] Steptoe, A., Pollard, T. M., & Wardle, J. (1995). Development of a measure of the motives underlying the selection of food: The Food Choice Questionnaire. Appetite, 25(3), 267–284. doi: 10.1006/appe.1995.0061

[5] Teodoro, M. A., Silva, W. R. da, Spexoto, M. C. B., & Silva Júnior, S. I. da. (2024). Factors of food choice and nutritional intake of Brazilian older adults according sociodemographic and health characteristics. Appetite, 199, 107379–107379. https://doi.org/10.1016/j.appet.2024.107379

[6] Smiliotopoulos, T., Magriplis, E., & Zampelas, A. (2020). Validation of a Food Propensity Questionnaire for the Hellenic National Nutrition and Health Survey (HNNHS) and Results on This Population's Adherence to Key Food-Group Nutritional Guidelines. *Nutrients*, *12*(6), 1808. https://doi.org/10.3390/nu12061808
