# Supplementary material for: Texture-Modified Food Attitudes and Behaviours Are Culturally Patterned in Community-Dwelling Older Adults
Source: Nutrients. 2026 Sep 30;18(19):3231. doi: 10.3390/nu18193231 (PMC13666978; doi:10.3390/nu18193231)
Supplement: Supplementary file 1 [file nutrients-18-03231-s001.zip › Table S1. Significant associations between nutrition-related knowledge, attitudes and behaviours (KAB) items and food-class preference.pdf]

Supplementary Table S1. Significant associations between nutrition-related knowledge, attitudes and behaviours (KAB) items and food-class preference.

KAB item	Domain	Outcome	Comparison	Estimate (SD units)	SE	P_{adj}	95% CI
Nutrient importance – calcium	Knowledge	Frequency	Indian vs Chinese	0.62	0.177	*0.012*	0.273, 0.967
Balanced diet importance	Knowledge	Liking	Western vs Chinese	0.426	0.136	*0.038*	0.159, 0.694
Nutrient importance – iron	Knowledge	Liking	Indian vs Chinese	0.385	0.119	*0.033*	0.153, 0.618
Nutrient importance – iron	Knowledge	Liking	Malay vs Chinese	0.325	0.091	*0.016*	0.147, 0.503
Balanced diet importance	Knowledge	Composite	Indian vs Chinese	0.322	0.059	*<0.001*	0.206, 0.438
Knowledge of texture-modified foods	Knowledge	Frequency	Malay vs Chinese	0.277	0.061	*0.002*	0.157, 0.397
Balanced diet importance	Knowledge	Liking	Sweet vs Savoury	0.274	0.089	*0.041*	0.099, 0.448
Balanced diet importance	Knowledge	Liking	Snack vs Main meal	0.268	0.082	*0.033*	0.107, 0.428
Knowledge of texture-modified foods	Knowledge	Frequency	Indian vs Chinese	0.254	0.08	*0.030*	0.097, 0.410
Balanced diet importance	Knowledge	Composite	Malay vs Chinese	0.203	0.047	*0.001*	0.110, 0.296
Knowledge of texture-modified foods	Knowledge	Composite	Indian vs Chinese	0.104	0.02	*<0.001*	0.066, 0.142
Knowledge of texture-modified foods	Knowledge	Composite	Malay vs Chinese	0.053	0.015	*0.011*	0.024, 0.081
Belief that TM food improves digestion	Knowledge	Liking	Sweet vs Savoury	0.042	0.013	*0.037*	0.016, 0.068
Belief that nutritional needs change with age	Knowledge	Composite	Indian vs Chinese	-0.078	0.025	*0.045*	-0.128, -0.029
Belief that TM food improves digestion	Knowledge	Frequency	Indian vs Chinese	-0.145	0.037	*0.004*	-0.218, -0.072
Knowledge of texture-modified foods	Knowledge	Liking	Western vs Chinese	-0.157	0.043	*0.014*	-0.242, -0.073
Nutrient importance – vitamin C	Knowledge	Liking	Western vs Chinese	-0.344	0.098	*0.018*	-0.536, -0.151
Nutrient importance – protein	Knowledge	Liking	Malay vs Chinese	-0.392	0.081	*<0.001*	-0.550, -0.233
Nutrient importance – omega-3	Knowledge	Frequency	Indian vs Chinese	-0.506	0.151	*0.020*	-0.803, -0.209

Nutrient importance – protein	Knowledge	Frequency	Malay vs Chinese	-0.529	0.148	*0.010*	-0.819, -0.239
Soft-food importance – aroma	Attitude	Frequency	Malay vs Chinese	0.527	0.107	*<0.001*	0.317, 0.736
Visual appeal – colour	Attitude	Frequency	Malay vs Chinese	0.456	0.121	*0.007*	0.220, 0.692
Belief that TM food improves overall eating	Attitude	Composite	Indian vs Chinese	0.294	0.068	*0.001*	0.160, 0.427
Reason for avoiding TM food – perceived as not nutritious	Attitude	Liking	Western vs Chinese	0.275	0.071	*0.009*	0.136, 0.414
Willingness to pay (general)	Attitude	Liking	Sweet vs Savoury	0.242	0.067	*0.015*	0.111, 0.373
Willingness to pay for TM food	Attitude	Liking	Sweet vs Savoury	0.242	0.076	*0.037*	0.092, 0.392
Belief that TM food improves overall eating	Attitude	Composite	Malay vs Chinese	0.207	0.052	*0.003*	0.106, 0.308
Visual appeal – colour	Attitude	Composite	Indian vs Chinese	0.148	0.038	*0.004*	0.074, 0.222
Reason for choosing TM food – nutritious	Attitude	Frequency	Malay vs Chinese	0.138	0.038	*0.009*	0.064, 0.213
Reason for avoiding TM food – perceived as not nutritious	Attitude	Liking	Snack vs Main meal	0.126	0.04	*0.038*	0.047, 0.206
Visual appeal – plating	Attitude	Composite	Western vs Chinese	0.121	0.033	*0.009*	0.056, 0.185
Reason for choosing TM food – personal liking	Attitude	Frequency	Indian vs Chinese	0.107	0.035	*0.039*	0.038, 0.177
Willingness to pay for TM food	Attitude	Composite	Sweet vs Savoury	0.105	0.034	*0.046*	0.038, 0.171
Reason for avoiding TM food – tedious preparation	Attitude	Liking	Indian vs Chinese	0.096	0.029	*0.033*	0.038, 0.153
Soft-food importance – appearance	Attitude	Composite	Snack vs Main meal	0.08	0.019	*0.002*	0.042, 0.117
Reason for choosing TM food – personal liking	Attitude	Frequency	Malay vs Chinese	0.076	0.023	*0.022*	0.031, 0.122
Reason for choosing TM food – ease of consumption	Attitude	Frequency	Malay vs Chinese	0.059	0.016	*0.007*	0.028, 0.089
Reason for choosing TM food – tasty	Attitude	Composite	Sweet vs Savoury	0.044	0.012	*0.010*	0.020, 0.067
Reason for choosing TM food – tasty	Attitude	Composite	Malay vs Chinese	0.042	0.014	*0.046*	0.015, 0.069
Reason for choosing TM food – personal liking	Attitude	Composite	Malay vs Chinese	0.018	0.005	*0.024*	0.007, 0.028

Reason for choosing TM food – fear of eating regular food	Attitude	Composite	Malay vs Chinese	0.017	0.004	*<0.001*	0.010, 0.025
Reason for avoiding TM food – not appetizing	Attitude	Composite	Malay vs Chinese	-0.036	0.01	*0.009*	-0.055, -0.017
Reason for avoiding TM food – tedious preparation	Attitude	Composite	Malay vs Chinese	-0.04	0.012	*0.026*	-0.063, -0.016
Reason for avoiding TM food – dislike of taste	Attitude	Composite	Malay vs Chinese	-0.048	0.013	*0.006*	-0.072, -0.023
Reason for choosing TM food – doctor's recommendation	Attitude	Liking	Malay vs Chinese	-0.057	0.018	*0.036*	-0.092, -0.022
Reason for avoiding TM food – tedious preparation	Attitude	Composite	Indian vs Chinese	-0.062	0.013	*<0.001*	-0.088, -0.036
Reason for avoiding TM food – uncertain about taste	Attitude	Frequency	Snack vs Main meal	-0.07	0.023	*0.038*	-0.114, -0.025
Soft-food importance – mouthfeel	Attitude	Composite	Snack vs Main meal	-0.07	0.022	*0.041*	-0.113, -0.026
Reason for choosing TM food – nutritious	Attitude	Liking	Sweet vs Savoury	-0.08	0.021	*0.011*	-0.121, -0.038
Reason for choosing TM food – nutritious	Attitude	Liking	Snack vs Main meal	-0.081	0.019	*0.004*	-0.119, -0.043
Soft-food importance – mouthfeel	Attitude	Composite	Sweet vs Savoury	-0.084	0.024	*0.015*	-0.131, -0.037
Intention to choose TM food if chewing becomes difficult	Attitude	Composite	Malay vs Chinese	-0.104	0.019	*<0.001*	-0.141, -0.067
Reason for avoiding TM food – dislike of texture	Attitude	Liking	Indian vs Chinese	-0.12	0.04	*0.044*	-0.198, -0.043
Soft-food importance – appearance	Attitude	Liking	Sweet vs Savoury	-0.143	0.047	*0.048*	-0.236, -0.050
Reason for avoiding TM food – tedious preparation	Attitude	Frequency	Malay vs Chinese	-0.149	0.048	*0.036*	-0.243, -0.055
Importance of TM food flavour familiarity	Attitude	Liking	Snack vs Main meal	-0.149	0.048	*0.038*	-0.243, -0.056
Reason for avoiding TM food – dislike of texture	Attitude	Liking	Western vs Chinese	-0.159	0.041	*0.009*	-0.240, -0.078
Soft-food importance – mouthfeel	Attitude	Liking	Indian vs Chinese	-0.328	0.089	*0.014*	-0.502, -0.153
Willingness to pay for TM food	Attitude	Frequency	Malay vs Chinese	-0.579	0.162	*0.010*	-0.895, -0.262

Importance of TM food flavour familiarity	Attitude	Frequency	Indian vs Chinese	-0.584	0.139	*0.002*	-0.856, -0.313
Frequency of TM consumption	Behaviour	Frequency	Indian vs Chinese	1.65	0.415	*0.004*	0.837, 2.463
Frequency of TM consumption	Behaviour	Frequency	Malay vs Chinese	1.124	0.283	*0.004*	0.570, 1.678
Importance of ease of chewing/swallowing in food choice	Behaviour	Frequency	Indian vs Chinese	0.653	0.156	*0.002*	0.348, 0.958
Importance of ease of chewing/swallowing in food choice	Behaviour	Frequency	Malay vs Chinese	0.503	0.12	*0.002*	0.267, 0.738
Number of main meals per day	Behaviour	Liking	Malay vs Chinese	0.472	0.114	*0.004*	0.249, 0.695
Use of oral nutritional supplements	Behaviour	Frequency	Indian vs Chinese	0.43	0.118	*0.009*	0.199, 0.662
Preference for home-cooked food	Behaviour	Liking	Western vs Chinese	0.396	0.128	*0.040*	0.145, 0.647
TM preparation challenge – lack of equipment	Behaviour	Frequency	Indian vs Chinese	0.393	0.088	*0.002*	0.221, 0.565
No food avoidance reported	Behaviour	Frequency	Malay vs Chinese	0.249	0.083	*0.044*	0.086, 0.411
Prior experience trying texture-modified foods	Behaviour	Frequency	Snack vs Main meal	0.183	0.057	*0.025*	0.072, 0.294
TM preparation challenge – lack of equipment	Behaviour	Liking	Western vs Chinese	0.126	0.037	*0.025*	0.054, 0.199
Prior experience trying texture-modified foods	Behaviour	Composite	Malay vs Chinese	0.121	0.02	*<0.001*	0.082, 0.161
Food avoidance – dairy	Behaviour	Frequency	Indian vs Chinese	0.121	0.029	*0.003*	0.064, 0.179
Prior experience trying texture-modified foods	Behaviour	Composite	Indian vs Chinese	0.096	0.027	*0.010*	0.044, 0.148
Food avoidance – dairy	Behaviour	Frequency	Malay vs Chinese	0.091	0.023	*0.004*	0.046, 0.137
Prior experience trying texture-modified foods	Behaviour	Composite	Sweet vs Savoury	0.078	0.017	*<0.001*	0.045, 0.111
Food avoidance – high sugar	Behaviour	Liking	Sweet vs Savoury	0.067	0.016	*0.004*	0.036, 0.098
Food avoidance – high fat	Behaviour	Liking	Snack vs Main meal	0.035	0.009	*0.009*	0.017, 0.053
Food avoidance – dairy	Behaviour	Liking	Snack vs Main meal	-0.035	0.01	*0.026*	-0.055, -0.015
Importance of ease of chewing/swallowing in food choice	Behaviour	Composite	Indian vs Chinese	-0.121	0.038	*0.034*	-0.194, -0.047

Importance of ease of chewing/swallowing in food choice	Behaviour	Composite	Malay vs Chinese	-0.144	0.03	*<0.001*	-0.202, -0.086
TM preparation challenge – uncertainty	Behaviour	Frequency	Indian vs Chinese	-0.246	0.074	*0.021*	-0.390, -0.101
Number of non-main-meal snacks per day	Behaviour	Liking	Malay vs Chinese	-0.472	0.096	*<0.001*	-0.660, -0.284

Results are from linear mixed-effects models adjusted for age, sex, ethnicity, and BMI, with random intercepts for participant and food item. Β coefficients represent KAB x food-class interaction effects, indicating the difference in the KAB-food-rating association between the named comparison class and its reference class. Positive β values indicate a weaker association in the comparison class. Benjamini-Hochberg-adjusted P values are reported. Additional significant interaction findings are reported here, while selected findings are highlighted in Table 4.
